# A genome-wide search for common SNP x SNP interactions on the risk of venous thrombosis

**DOI:** 10.1186/1471-2350-14-36

**Published:** 2013-03-20

**Authors:** Nicolas Greliche, Marine Germain, Jean-Charles Lambert, William Cohen, Marion Bertrand, Anne-Marie Dupuis, Luc Letenneur, Mark Lathrop, Philippe Amouyel, Pierre-Emmanuel Morange, David-Alexandre Trégouët

**Affiliations:** 1 INSERM, UMR_S 937; Institute of Cardiometabolism And Nutrition (ICAN), Université Pierre et Marie Curie Paris 6, Paris F-75013, France; 2 INSERM, UMR_S 744; Institut Pasteur de Lille, Université de Lille Nord de France, Lille F-59019, France; 3 INSERM, UMR_S 1062, Aix-Marseille Université, Marseille F-13385, France; 4 INSERM, UMR_S 708, Université Pierre et Marie Curie, Paris F-75013, France; 5 INSERM, UMR_S 888, F-34093, Univ Montpellier, Montpellier F-34093, France; 6 INSERM, U897, Univ. Bordeaux, ISPED, Bordeaux, F-33000, France; 7 Commissariat à l’Energie Atomique, Institut de Génomique, Centre National de Génotypage, Evry, F-91057, France; 8 Centre Hospitalier Régional Universitaire de Lille, Lille F-59037, France

**Keywords:** Venous thrombosis, Genome-Wide Association Study, Interaction, Factor VIII

## Abstract

**Background:**

Venous Thrombosis (VT) is a common multifactorial disease with an estimated heritability between 35% and 60%. Known genetic polymorphisms identified so far only explain ~5% of the genetic variance of the disease. This study was aimed to investigate whether pair-wise interactions between common single nucleotide polymorphisms (SNPs) could exist and modulate the risk of VT.

**Methods:**

A genome-wide SNP x SNP interaction analysis on VT risk was conducted in a French case–control study and the most significant findings were tested for replication in a second independent French case–control sample. The results obtained in the two studies totaling 1,953 cases and 2,338 healthy subjects were combined into a meta-analysis.

**Results:**

The smallest observed p-value for interaction was p = 6.00 10^-11^ but it did not pass the Bonferroni significance threshold of 1.69 10^-12^ correcting for the number of investigated interactions that was 2.96 10^10^. Among the 37 suggestive pair-wise interactions with p-value less than 10^-8^, one was further shown to involve two SNPs, rs9804128 (*IGFS21* locus) and rs4784379 (*IRX3* locus) that demonstrated significant interactive effects (p = 4.83 10^-5^) on the variability of plasma Factor VIII levels, a quantitative biomarker of VT risk, in a sample of 1,091 VT patients.

**Conclusion:**

This study, the first genome-wide SNP interaction analysis conducted so far on VT risk, suggests that common SNPs are unlikely exerting strong interactive effects on the risk of disease.

## Background

Venous Thrombosis (VT) is a common complex disease affecting ~0.2% of individuals a year. VT includes deep vein thrombosis and pulmonary embolism, the latter being characterized by a one year mortality rate of ~10% excluding patients with malignancies [1]. As a complex trait, VT is considered as resulting from the interplay between environmental and genetic factors, that could interact with each other, to modulate VT risk [2,3]. The recent Genome Wide Association Studies (GWAS) strategy brought great hopes to identify novel susceptibility loci to human diseases and some true successes were obtained in the field of VT genetics. Novel genes recently identified to harbor common susceptibility alleles (i.e. with allele frequency > 0.05) for VT include *GP6*, *HIVEP1*, *KNG1*, *STAB2*, *STXBP5* and *VWF* (reviewed in [4]). However, none of the identified risk alleles demonstrated genetic effects stronger than those of the established VT-associated genes known before the GWAS era, *ABO*, *F2*, *F5* and *FGG*[5]. As for most multifactorial diseases, risk alleles for VT identified so far only explain a small proportion of the familial risk of disease [6]. Alternative strategies are needed to identify the army sources that could contribute to the unexplained heritability and these include gene-gene and gene-environment interactions, deep sequencing, transcriptomic analyses and epigenomics [7-10].

In this work, we were interested in assessing whether interaction between common polymorphisms could contribute to VT risk. To our knowledge, studies that have investigated this hypothesis were mainly dedicated to known candidate genes [11,12] and no attempt has been made to address it without any *a priori* hypothesis. This is why, we here take advantage of the large amount of genetic information we have collected through two French GWAS on VT [6,13] to conduct the first genome-wide search for SNP x SNP interaction with respect to VT risk.

## Methods

This work was based on two French GWAS on VT, the Early-Onset Venous Thrombosis (EOVT) and the Marseille Thrombosis Association (MARTHA) studies. These two studies have already been extensively described in [5,6,14] for EOVT and in [6,15-17] for MARTHA.

### Ethical approval

Each individual study was approved by its institutional ethics committee and informed written consent was obtained in accordance with the Declaration of Helsinki. Ethics approval were obtained from the “Departement santé de la direction générale de la recherche et de l’innovation du ministère” (Projects DC: 2008-880 & 09.576) and from the institutional ethics committees of the Kremlin-Bicetre Hospital.

### Studied populations and phenotype measurements

Briefly, in both studies, VT patients were cases, with a documented history of VT and free of well known strong genetic risk factors including antithrombin (AT), protein C (PC) or protein S (PS) deficiency, homozygosity for FV Leiden or F2 20210A mutations and lupus anticoagulant. In EOVT, patients were selected to experience idiopathic VT before the age of 50. Controls were French individuals selected from two healthy populations, SUVIMAX [18] and the Three City Study [19], for EOVT and MARTHA, respectively. The EOVT case–control study included 411 patients and 1,228 healthy subjects, while MARTHA was composed of 1,542 patients and 1,110 healthy subjects, all the individuals being of European origin, with the majority being of French descent. A summary of the population characteristics is provided in Additional file 1.

Several key quantitative biomarkers of VT risk have been measured in MARTHA patients. The detailed description of the corresponding measurements has been previously described in [15] for AT, PC, PS and the agkistrodon contortrix venom (ACV) test that explores the PC pathway, in [17] for Factor VIII (FVIII) and von Willebrand Factor (VWF), and in [16] for Activated Partial Thromboplastin Time (aPTT) and Prothrombin Time (PT).

### Genotyping

Individuals participating in the EOVT study were genotyped for 317,139 SNPs using the Illumina Sentrix HumanHap300 Beadchip. The application of the quality control criteria described in [5] led the final selection of 291,872 autosomal SNPs for analysis. As detailed in [6], individuals participating to the MARTHA GWAS were typed with the Illumina Human 610-Quad and Human660W-Quad Beadchips. 481,002 autosomal SNPs remained for analysis after quality control.

### Statistical analysis

Our search for genome wide interactions was conducted in two steps. A first screening for pairwise SNPs interactions was carried out in the EOVT study. The first part of this discovery screening consisted in reducing redundancy between SNPs by keeping only one SNP out of all SNPs in strong pairwise linkage disequilibrium (r^2^ > 0.90) within a window of 50 kb. Pairwise SNPs interactions were then tested by a logistic regression analysis where both SNPs were coded under an additive model (0,1 and 2 according to the number of rare alleles) and an interaction term was added in the model. For this, we used the plink software [20]. All interactions significant at p < 10^-4^ were further assessed at a second step in the larger MARTHA study. When SNPs were not available in the latter sample, the best available proxy in term of r^2^, according to the SNAP database [21], was used. The same logistic regression model was applied in the MARTHA study. Results obtained in the two GWAS were then meta-analyzed through a fixed-effect model relying on the inverse-variance weighting as implemented in the METAL software (http://www.sph.umich.edu/csg/abecasis/metal). Homogeneity of associations across the two GWAS studies was tested using the Mantel-Haenszel method [22].

The most significant interactions were then further assessed in relation to quantitative biomarkers of VT risk in MARTHA patients. For this, standard linear regression analyses were conducted with the same additive allele coding as for the binary trait analysis. Analyses were adjusted for age, sex and ABO blood group. For AT, PC, PS and ACV, individuals under anticoagulant were excluded. The THESIAS software [23] was used to illustrate the detected pairwise SNP interactions.

## Results and discussion

We first applied a pairwise tagging approach to discard redundant SNPs using a r^2^ threshold of 0.90, that led to the final selection of 243,189 SNPs from the EOVT study.

2.96 10^10^ pairwise SNPs interactions were then tested in EOVT, but none of them reached the Bonferroni corrected p-value of 1.69 10^-12^. Nevertheless, all interactions with p-value less than 10^-4^ (n = 2,126,084) were further assessed in MARTHA. The smallest observed p-value was 6.73 10^-7^, but it did not pass the Bonferroni correction (p < 2.35 10^-8^) for the number of interactions tested at this second step.

The meta-analysis of the results obtained in EOVT and MARTHA led to 37 suggestive interactions with p-values lower than 10^-8^ and with consistent effects in both studies (Table 1). The smallest one, p = 6.00 10^-11^, was observed for two SNPs in the vicinity of *SURF6* gene that is ~40 kb from the *ABO* locus. After adjusting for the ABO blood group, this interaction vanished (p = 0.37) suggesting that this interaction had captured the ABO effect through the linkage disequilibrium extending at this locus.

**Table 1:** **Pairwise SNP interactions with suggestive statistical evidence (p < 10**^**-8**^**) in the meta-analysis of two GWAS datasets gathering 1,953 cases and 2,338 controls**

						**EOVT**				**MARTHA**				**Combined**				
**SNP1 X SNP2**						**MAF**^**(2)**^		**Interaction**		**MAF**			**Interaction**					
**rsID**	**Alleles**^**(1)**^	**CHR**	**rsID**	**Alleles**	**CHR**	**SNP1**	**SNP2**	**OR**^**(3)**^	**P**	**SNP1**	**SNP2**	**OR**	**P**	**OR **^**(4)**^	**P**	**Cochran Q**^**(5)**^	**I**^**2**^	**P**^**(6)**^
rs493014	T/G	9	rs886090	G/A	9	0.31	0.33	1.72	1.85 × 10^-5^	0.30	0.31	1.6	6.73 × 10^-7^	1.64	6.00 × 10^-11^	0.21	0%	0.65
rs1336472	G/A	1	rs4715555	A/G	6	0.41	0.39	1.64	4.10 × 10^-5^	0.40	0.38	1.49	2.00 × 10^-6^	1.54	4.24 × 10^-10^	0.42	0%	0.52
rs380904	G/A	8	rs8086028	G/A	18	0.30	0.27	1.96	3.76 × 10^-6^	0.29	0.31	1.55	1.12 × 10^-5^	1.67	4.51 × 10^-10^	1.79	44%	0.18
rs6815916	A/G	4	rs6092326	C/T	20	0.09	0.48	2.37	4.32 × 10^-5^	0.09	0.47	1.98	2.95 × 10^-6^	2.10	6.84 × 10^-10^	0.51	0%	0.48
rs2282015	T/G	10	rs13050454	G/A	21	0.41	0.43	1.81	3.52 × 10^-7^	0.41	0.42	1.37	7.68 × 10^-5^	1.50	8.36 × 10^-10^	3.90	74%	0.05
rs7648704	T/G	3	rs4868644	C/T	5	0.33	0.49	1.64	7.36 × 10^-5^	0.33	0.49	1.52	2.88 × 10^-6^	1.56	9.89 × 10^-10^	0.26	0%	0.61
rs1985317	T/C	9	rs827637	G/A	10	0.39	0.46	0.55	7.13 × 10^-7^	0.41	0.46	0.72	7.73 × 10^-5^	0.66	1.32 × 10^-9^	3.42	71%	0.06
rs2321744	A/G	13	rs6497540	T/G	16	0.09	0.41	0.43	8.61 × 10^-5^	0.1	0.42	0.52	2.98 × 10^-6^	0.49	1.38 × 10^-9^	0.55	0%	0.46
rs315122	T/G	12	rs884483	T/C	15	0.29	0.11	2.61	1.92 × 10^-5^	0.31	0.12	1.87	7.90 × 10^-6^	2.05	1.42 × 10^-9^	1.59	37%	0.21
rs1423386	A/G	5	rs6491679	T/G	13	0.2	0.29	1.92	7.24 × 10^-5^	0.2	0.29	1.66	4.17 × 10^-6^	1.73	1.63 × 10^-9^	0.56	0%	0.45
rs7714670	T/C	5	rs12880735	G/A	14	0.44	0.34	1.75	4.59 × 10^-6^	0.44	0.36	1.42	3.32 × 10^-5^	1.52	1.75 × 10^-9^	1.99	50%	0.16
rs9392653	C/T	6	rs7780976	A/C	7	0.27	0.18	2.14	2.28 × 10^-6^	0.29	0.19	1.57	5.49 × 10^-5^	1.74	1.83 × 10^-9^	2.47	60%	0.12
rs9804128	A/G	1	rs4784379	G/A	16	0.27	0.25	1.97	2.73 × 10^-5^	0.26	0.24	1.60	9.45 × 10^-6^	1.71	1.90 × 10^-9^	1.14	13%	0.28
rs1364505	G/A	7	rs1204660	G/A	20	0.30	0.16	2.14	2.32 × 10^-5^	0.33	0.16	1.67	1.11 × 10^-5^	1.80	2.10 × 10^-9^	1.35	26%	0.25
rs2288073	A/G	2	rs10771022	G/T	12	0.30	0.34	1.71	7.94 × 10^-5^	0.29	0.34	1.55	5.51 × 10^-6^	1.60	2.11 × 10^-9^	0.35	0%	0.55
rs1367228	C/A	2	rs3905075	C/T	13	0.43	0.41	1.61	9.44 × 10^-5^	0.45	0.4	1.44	4.22 × 10^-6^	1.49	2.20 × 10^-9^	0.62	0%	0.43
rs536477	G/A	1	rs1937920	A/G	10	0.43	0.26	0.57	3.27 × 10^-5^	0.43	0.27	0.67	1.40 × 10^-5^	0.63	2.93 × 10^-9^	0.90	0%	0.34
rs2710201	A/G	7	rs3780293	G/A	9	0.06	0.34	0.35	6.84 × 10^-5^	0.06	0.36	0.43	9.92 × 10^-6^	0.40	3.30 × 10^-9^	0.38	0%	0.54
rs12541254	G/A	8	rs305009	G/A	15	0.35	0.23	1.99	3.15 × 10^-6^	0.34	0.23	1.50	7.63 × 10^-5^	1.65	3.33 × 10^-9^	2.39	58%	0.12
rs4507975	A/G	1	rs9914518	G/A	17	0.29	0.46	0.61	9.59 × 10^-5^	0.29	0.47	0.67	7.95 × 10^-6^	0.65	3.58 × 10^-9^	0.32	0%	0.57
rs2771051	T/G	9	rs827637	G/A	10	0.37	0.46	0.52	9.27 × 10^-8^	0.37	0.46	0.75	4.59 × 10-4	0.67	3.82 × 10^-9^	6.08	84%	0.01
rs10516089	T/C	5	rs11072930	T/C	15	0.32	0.28	0.51	2.66 × 10^-6^	0.31	0.3	0.69	7.19 × 10^-5^	0.63	3.86 × 10^-9^	3.12	68%	0.08
rs10504130	G/A	8	rs2847351	A/G	18	0.15	0.3	2.46	1.04 × 10^-5^	0.14	0.32	1.69	3.07 × 10^-5^	1.88	4.46 × 10^-9^	2.40	58%	0.12
rs318497	G/A	6	rs7019259	A/G	9	0.49	0.07	0.29	2.56 × 10^-6^	0.49	0.07	0.51	8.40 × 10^-5^	0.43	4.54 × 10^-9^	3.21	69%	0.07
rs6695223	T/C	1	rs1763510	C/T	6	0.12	0.39	2.49	6.00 × 10^-6^	0.13	0.39	1.66	4.31 × 10^-5^	1.86	4.70 × 10^-9^	2.91	66%	0.09
rs1336708	A/G	13	rs1423386	A/G	5	0.26	0.2	0.51	6.77 × 10^-5^	0.25	0.2	0.61	1.20 × 10^-5^	0.58	4.85 × 10^-9^	0.79	0%	0.37
rs6771316	G/A	3	rs10986432	T/C	9	0.14	0.18	2.41	4.64 × 10^-5^	0.13	0.17	1.99	2.20 × 10^-5^	2.13	5.26 × 10^-9^	0.50	0%	0.48
rs664910	A/G	3	rs877228	G/A	15	0.30	0.47	1.63	6.05 × 10^-5^	0.30	0.44	1.44	1.92 × 10^-5^	1.50	6.63 × 10^-9^	0.71	0%	0.40
rs9945428	C/A	18	rs4823535	G/A	22	0.30	0.28	0.58	7.47 × 10^-5^	0.30	0.26	0.65	1.85 × 10^-5^	0.62	6.88 × 10^-9^	0.46	0%	0.50
rs1910358	T/C	5	rs9981595^(7)^	T/G	21	0.23	0.12	2.21	9.60 × 10^-5^	0.23	0.11	1.93	1.63 × 10^-5^	2.03	7.14 × 10^-9^	0.30	0%	0.59
rs6771725	G/T	3	rs10507246	G/T	12	0.26	0.08	2.6	4.02 × 10^-5^	0.28	0.09	2.04	3.77 × 10^-5^	2.22	8.60 × 10^-9^	0.71	0%	0.40
rs16865717	C/T	2	rs2009579	C/T	20	0.27	0.36	1.9	5.22 × 10^-6^	0.29	0.36	1.43	9.59 × 10^-5^	1.56	8.82 × 10^-9^	2.88	65%	0.09
rs2028385	A/G	12	rs2038227	A/C	16	0.16	0.39	2.19	3.36 × 10^-7^	0.16	0.37	1.47	7.11 × 10^-4^	1.69	8.82 × 10^-9^	4.40	77%	0.04
rs10476160	A/G	5	rs1707420	C/T	8	0.21	0.48	0.56	6.35 × 10^-5^	0.20	0.48	0.65	2.48 × 10^-5^	0.62	9.09 × 10^-9^	0.75	0%	0.39
rs971572	C/A	1	rs10828151	A/C	10	0.32	0.07	0.35	3.43 × 10^-5^	0.32	0.07	0.47	4.38 × 10^-5^	0.42	9.30 × 10^-9^	0.88	0%	0.35
rs6858430	C/T	4	rs4800250	A/G	18	0.20	0.40	1.86	2.44 × 10^-5^	0.21	0.40	1.52	5.16 × 10^-5^	1.62	9.67 × 10^-9^	1.30	23%	0.25
rs467650	T/C	5	rs7153749	T/C	14	0.36	0.44	0.59	1.69 × 10^-5^	0.37	0.44	0.71	6.00 × 10^-5^	0.67	9.91 × 10^-9^	1.75	43%	0.19

(1) Common/minor alleles.

(2) Minor Allele Frequency.

(3) Odds ratio for VT associated with the interaction of the two minor alleles under a logistic model assuming additive allelic effect.

(4) Pooled Odds ratio derived from a fixed-effect model analysis using the inverse-variance method as implemented in METAL. None of the reported interactions demonstrated evidence for heterogeneity across GWAS samples after Bonferroni correction (p > 0.05/37 for all homogeneity test pvalues).

(5) Cochran Q statistics assessing the extend of heterogeneity across the two GWAS samples.

(6) P value of the Mantel-Haenszel statistic assessing heterogeneity across GWAS samples.

(7) rs2836978 serves as a proxy (r^2^ = 1) for rs9981595 in the discovery GWAS.

Despite the lack of study-wise statistical interactions, we could not exclude that some genuine interaction phenomena hide in the list of suggestive interactions (Table 1). We hypothesized that the use of additional biological information on quantitative biomarkers of VT risk could help in digging into this list. We therefore investigated whether the identified interactive SNPs could exert their effect on VT biomarkers available in MARTHA: ACV, aPTT, AT, Fibrinogen, FVIII, PC, PS, PT and VWF. At the Bonferroni threshold of 1.50 10^-4^ for the number of performed tests (i.e. 333 = 37 SNPs x 9 phenotypes ), one interaction was statistically significant (p = 4.82 10^-5^). It involved rs9804128 lying in the promoter region of the *IGSF21* gene and the rs4784379 mapping 130 kb downstream the *IRX3* locus, the two SNPs interacting to modulate plasma FVIII levels. As shown in Table 2, carriers of the rs9804128-G and rs4784379-A alleles were associated with the highest plasma FVIII levels compared to the three other alleles combinations. At contrast, these individuals were associated with ~2 fold decreased in VT risk, the frequency of the GA combination being 8.3% in controls and 4.6% in patients (Table 2). Looking deeply to the diplotypes formed by these two SNPs revealed that patients carrying without any ambiguity the GA combination, ie those carrying either the rs9804128-GG genotype and the rs4784379-A allele or the rs9804128-GA genotype and the rs4784379-AA genotype, exhibited the highest plasma FVIII levels (Table 3). Individuals ambiguous for the GA combination, who are those heterozygotes at both rs9804128 and rs4784379, were at intermediate FVIII levels (Table 3).

**Table 2:** Interactive effects of the rs9804128 and rs4784379 on the risk of VT and on plasma FVIII levels

		**EOVT**		**MARTHA**		**Combined**		**MARTHA patients**^**(1)**^	
		**Frequency**		**Frequency**		**Frequency**		**Frequency**	**Haplotypic FVIII expected mean [95%CI]**
**rs9804128**	**rs4784379**	**Controls**	**Cases**	**Controls**	**Cases**	**Controls**	**Cases**		
		**N = 1,228**	**N = 411**	**N = 1,110**	**N = 1,542**	**N = 2,338**	**N = 1953**		
A	G	0.561	0.530	0.579	0.551	0.569	0.547	0.548	68.77 [66.27–71.26]
A	A	0.169	0.198	0.165	0.185	0.167	0.188	0.184	62.34 [58.03–66.64]
G	G	0.190	0.237	0.170	0.214	0.181	0.219	0.220	62.09 [56.35–67.83]
G	A	0.080	0.035	0.085	0.050	0.083	0.046	0.048	91.95 [92.98–100.9]
		p^(2)^ = 2.73 10^-5^			p^(2)^ = 9.45 10^-6^					p^(3)^ = 1.90 10^-9^	p^(4)^ = 6.89 10^-5^

^(1)^ In MARTHA, 1091 patients were measured for FVIII levels.

^(2)^ p-value of the interaction term between the two SNPs in the logistic regression analysis under the assumption of additive allele effects.

^(3)^ p-value obtained from the meta-analysis of the EOVT and MARTHA samples using a fixed-effect model.

^(4)^ p-value of the interaction term between the two SNPs in the linear regression analysis, adjusted for age, sex, ABO blood group and F5/F2 carriers mutations.

**Table 3:** Plasma FVIII levels according to the rs9804128 and rs4784379 polymorphisms in 1091 VT patients

		**rs4784379**	
rs9804128	AA	AG	GG
AA	115.91 (32.80)	132.70 (49.75)	136.16 (51.35)
	N = 34	N = 231	N = 321
GA	155.93 (77.17)	141.42 (56.03)	131.76 (47.11)
	N = 16	N = 144	N = 266
GG	156.00 (68.98)	150.17 (42.90)	122.90 (60.11)
	N = 4	N = 23	N = 52

Mean (SE) are shown.

To our knowledge, this work is the first attempt in the field of VT genetics to investigate, at the genome-wide scale, the presence of interactive effects derived from common SNPs. This study did not detect interactions that reached the Bonferroni correction for the number of investigated interactions. The absence of such interaction could of course be due to low power. According to the distributions of the minor allele frequencies and the marginal allelic effects observed in the EOVT study, we computed the minimum OR for interaction that could be detectable with a 80% power [24,25]. These calculations suggest that our discovery cohort was only well powered to detect interactive ORs greater than 2.8 at the genome-wide statistical level of 1.69 10^-12^ and ORs greater than 1.8 at the p <10^-4^ threshold [Additional file 2]. The power to detect in our second sample the most significant observed interactions was about 50% [24,25]. As a consequence, despite the use of two large GWAS datasets on VT, this study was not powerful enough to detect interactions between common SNPS characterized by interactive ORs smaller than ~2.

There is still no consensus about the most efficiency way to perform a genome-wide search for SNP x SNP interaction. A plethora of statistical methods are applicable to the detection of such interactions eg [8,26-29] and none of them could be considered as the panacea. Comparing the performances of different methodologies is of great importance but out of the scope of this manuscript. We rather focused in the present work on the application of a standard methodology, the logistic regression model, that has been shown to be a valid methodology for detecting interaction between SNPs [8]. Different strategies can still be adopted within the logistic regression framework. Some people advocate to restrict the search for interaction to the set of most “significant” SNPs observed in single locus analysis. However, in that case, which statistical threshold should be used for selecting SNPs with significant marginal associations? Nevertheless, we further confined our search for interaction to SNPs with statistical evidence for association in univariate analysis as low as p < 10^-3^ or p < 0.05. We did not identify pair-wise significant interaction that were homogeneous between EOVT and MARTHA, and that satisfied the relevant Bonferroni correction (data not shown). Others suggest to use external biological information to refine the research strategy. Pathway-based analysis focusing only on the pairwise interactions between candidate gene SNPs could be such a strategy. By focusing only on SNPs mapping the VT candidate genes listing in the Supplementary Table 1 in [6], we did not detect any Bonferroni-corrected significant interaction that replicate in the EOVT and MARTHA study (data not shown). Another possibility could consist in assessing whether the most promising interactive effects could also be observed on quantitative traits known to be associated with the disease. Doing so, we observed that the rs9804128 and rs4784379 could interact to modulate both the risk of VT and the variability of FVIII levels. The rs9804128 lies in the proximal promoter of the *IGFS21* gene and, according to the SNAP database [21], it is not in strong LD (r^2^ > 0.8) with any other SNP. Conversely, the rs4784379 is in strong LD with several SNPs, all located at least 100 kb away from the *IRX3* locus. However, the observed interaction could be considered as counterintuitive since the allele combination associated with increased FVIII levels was found less frequent in cases than in controls. This phenomenon could nevertheless be observed in presence of a mortality bias when patients with high levels of FVIII levels are at a higher risk of VT-associated mortality (eg. pulmonary embolism) and then under-represented in the cases sample. Further investigations are needed to replicate this association that involved SNPs at genes on which very little is known with respect to VT.

## Conclusion

In conclusion, our work suggests that strong interactive (~OR >2) phenomena between common SNPs are unlikely to contribute much to the risk of the VT.

### Consent

Written informed consent was obtained from the patient for publication of this report and any accompanying images.

## Competing interests

The authors declare they have no competing interests.

## Authors’ contribution

NG and DAT carried out statistical analyses. MG, JCL and WC were responsible for data collection and database management. AMD, DAT, MB, ML, PA and PEM contributed to the study design whose direct implementation was coordinated by DAT and PEM. All authors read and approved the final manuscript.

## Pre-publication history

The pre-publication history for this paper can be accessed here:

http://www.biomedcentral.com/1471-2350/14/36/prepub

## Supplementary Material

Additional file 1 Summary characteristics of the two studied GWAS populations. Click here for file

Additional file 2 **Density of the minimal interactive Odds Ratio that can be detected with a 80% power in the EOVT study.** In green is shown the density distribution for a statistical level of 10^-4^.In black is the corresponding distribution for the genome-wide Bonferroni statistical level of 1.69 10^-12^. The mode of these distributions were 1.84 and 2.83, respectively. By symmetry on the logarithmic scale, only positive ORs are shown. Click here for file

## References

[B1] White RH The epidemiology of venous thromboembolism Circulation 2003 107 I4 I8 12814979 10.1161/01.CIR.0000078468.11849.66

[B2] Rosendaal FR Venous thrombosis: a multicausal disease Lancet 1999 353 1167 1173 10.1016/S0140-6736(98)10266-0 10209995

[B3] Souto JC Almasy L Borrell M Blanco-Vaca F Mateo J Soria JM Coll I Felices R Stone W Fontcuberta J Blangero J Genetic susceptibility to thrombosis and its relationship to physiological risk factors: the GAIT study. Genetic Analysis of Idiopathic Thrombophilia Am J Hum Genet 2000 67 1452 1459 10.1086/316903 11038326 PMC1287922

[B4] Morange PE Tregouet DA Lessons from genome-wide association studies in venous thrombosis J Thromb Haemost 2011 9 Suppl 1 258 264 21781262 10.1111/j.1538-7836.2011.04311.x

[B5] Tregouet DA Heath S Saut N Biron-Andreani C Schved JF Pernod G Galan P Drouet L Zelenika D Juhan-Vague I Common susceptibility alleles are unlikely to contribute as strongly as the FV and ABO loci to VTE risk: results from a GWAS approach Blood 2009 113 5298 5303 10.1182/blood-2008-11-190389 19278955

[B6] Germain M Saut N Greliche N Dina C Lambert JC Perret C Cohen W Oudot-Mellakh T Antoni G Alessi MC Genetics of venous thrombosis: insights from a new genome wide association study PLoS One 2011 6 e25581 10.1371/journal.pone.0025581 21980494 PMC3181335

[B7] Morange PE Tregouet DA Deciphering the molecular basis of venous thromboembolism: where are we and where should we go? Br J Haematol 2010 148 495 506 10.1111/j.1365-2141.2009.07975.x 19912223

[B8] Cordell HJ Detecting gene-gene interactions that underlie human diseases Nat Rev Genet 2009 10 392 404 19434077 10.1038/nrg2579 PMC2872761

[B9] Manolio TA Collins FS Cox NJ Goldstein DB Hindorff LA Hunter DJ McCarthy MI Ramos EM Cardon LR Chakravarti A Finding the missing heritability of complex diseases Nature 2009 461 747 753 10.1038/nature08494 19812666 PMC2831613

[B10] Eichler EE Flint J Gibson G Kong A Leal SM Moore JH Nadeau JH Missing heritability and strategies for finding the underlying causes of complex disease Nat Rev Genet 2011 11 446 450 20479774 10.1038/nrg2809 PMC2942068

[B11] Auro K Alanne M Kristiansson K Silander K Kuulasmaa K Salomaa V Peltonen L Perola M Combined effects of thrombosis pathway gene variants predict cardiovascular events PLoS Genet 2007 3 e120 10.1371/journal.pgen.0030120 17677000 PMC1934395

[B12] Pomp ER Doggen CJ Vos HL Reitsma PH Rosendaal FR Polymorphisms in the protein C gene as risk factor for venous thrombosis Thromb Haemost 2009 101 62 67 19132190

[B13] Tregouet DA Konig IR Erdmann J Munteanu A Braund PS Hall AS Grosshennig A Linsel-Nitschke P Perret C DeSuremain M Genome-wide haplotype association study identifies the SLC22A3-LPAL2-LPA gene cluster as a risk locus for coronary artery disease Nat Genet 2009 41 283 285 10.1038/ng.314 19198611

[B14] Smith NL Heit JA Tang W Teichert M Chasman DI Morange PE Genetic variation in F3 (tissue factor) and the risk of incident venous thrombosis: meta-analysis of eight studies J Thromb Haemost 2012 10 719 722 10.1111/j.1538-7836.2012.04665.x 22340074 PMC3397243

[B15] Oudot-Mellakh T Cohen W Germain M Saut N Kallel C Zelenika D Lathrop M Tregouet DA Morange PE Genome wide association study for plasma levels of natural anticoagulant inhibitors and protein C anticoagulant pathway: the MARTHA project Br J Haematol 2012 157 230 239 10.1111/j.1365-2141.2011.09025.x 22443383

[B16] Tang W Schwienbacher C Lopez LM Ben-Shlomo Y Oudot-Mellakh T Johnson AD Samani NJ Basu S Gogele M Davies G Genetic Associations for Activated Partial Thromboplastin Time and Prothrombin Time, their Gene Expression Profiles, and Risk of Coronary Artery Disease Am J Hum Genet 2012 91 152 162 22703881 10.1016/j.ajhg.2012.05.009 PMC3397273

[B17] Antoni G Oudot-Mellakh T Dimitromanolakis A Germain M Cohen W Wells P Lathrop M Gagnon F Morange PE Tregouet DA Combined analysis of three genome-wide association studies on vWF and FVIII plasma levels BMC Med Genet 2011 12 102 21810271 10.1186/1471-2350-12-102 PMC3163514

[B18] Hercberg S Galan P Preziosi P Bertrais S Mennen L Malvy D Roussel AM Favier A Briancon S The SU.VI.MAX Study: a randomized, placebo-controlled trial of the health effects of antioxidant vitamins and minerals Arch Intern Med 2004 164 2335 2342 10.1001/archinte.164.21.2335 15557412

[B19] 3C Study Group Vascular factors and risk of dementia: design of the Three-City Study and baseline characteristics of the study population Neuroepidemiology 2003 22 316 325 14598854 10.1159/000072920

[B20] Purcell S Neale B Todd-Brown K Thomas L Ferreira MA Bender D Maller J Sklar P de Bakker PI Daly M Sham PC PLINK: a tool set for whole-genome association and population-based linkage analyses Am J Hum Genet 2007 81 559 575 10.1086/519795 17701901 PMC1950838

[B21] Johnson AD Handsaker RE Pulit SL Nizzari MM O’Donnell CJ de Bakker PI SNAP: a web-based tool for identification and annotation of proxy SNPs using HapMap Bioinformatics 2008 24 2938 2939 10.1093/bioinformatics/btn564 18974171 PMC2720775

[B22] Mantel N Haenszel W Statistical aspects of the analysis of data from retrospective studies of disease J Natl Cancer Inst 1959 22 719 748 13655060

[B23] Tregouet DA Garelle V A new JAVA interface implementation of THESIAS: testing haplotype effects in association studies Bioinformatics 2007 23 1038 1039 10.1093/bioinformatics/btm058 17308338

[B24] Gauderman WJ Sample size requirements for association studies of gene-gene interaction Am J Epidemiol 2002 155 478 484 10.1093/aje/155.5.478 11867360

[B25] Demidenko E Sample size and optimal design for logistic regression with binary interaction Stat Med 2008 27 36 46 10.1002/sim.2980 17634969

[B26] Gyebesei A Moody J Semple CAM Haley CS Wei WH High-throughput analysis of epistasis in genome-wide association studies with BiForce Bioninformatics 2012 28 1957 1964 10.1093/bioinformatics/bts304 PMC3400955 22618535

[B27] Ueki M Cordell HJ Improved statistics for genome-wide interaction analysis PLoS Genet 2012 8 e1002625 10.1371/journal.pgen.1002625 22496670 PMC3320596

[B28] Hsu L Jiao S Dai JY Hutter C Peter U Kooperberg C Powerful cocktail methods for detecting genome-wide gene-environment interaction Genet Epidemiol 2012 36 183 194 10.1002/gepi.21610 22714933 PMC3654520

[B29] Van Steen K Travelling the world of gene-gene interactions Brief Bioinform 2012 13 1 19 10.1093/bib/bbr012 21441561

