# Supplementary material for: A genome-wide search for common SNP x SNP interactions on the risk of venous thrombosis
Source: BMC Med Genet. 2013 Mar 20;14:36. doi: 10.1186/1471-2350-14-36 (PMC3607886; doi:10.1186/1471-2350-14-36)
Supplement: Additional file 1 — Summary characteristics of the two studied GWAS populations. [file 1471-2350-14-36-S1.docx]

**Additional File1 Summary characteristics of the two studied GWAS populations**

|  | EOVT | | MARTHA | |
| --- | --- | --- | --- | --- |
|  | Controls | Cases | Controls | Cases |
|  | N = 1,228 | N = 411 | N = 1,110 | N = 1,542 |
| Gender (Male%) | 30% | 55% | 31% | 34% |
| Age (years)^a^ | 50 (6) | 36 (9) | 68 (2) | 47 (15) |
| FII 20210A carriers | - | 7% | - | 14% |
| FV Leiden carriers | - | 18% | - | 22% |

^a^ Mean (Standard Deviation)
