# Supplementary material for: A genome-wide search for common SNP x SNP interactions on the risk of venous thrombosis
Source: BMC Med Genet. 2013 Mar 20;14:36. doi: 10.1186/1471-2350-14-36 (PMC3607886; doi:10.1186/1471-2350-14-36)
Supplement: Additional file 2 — Density of the minimal interactive Odds Ratio that can be detected with a 80% power in the EOVT study. In green is shown the density distribution for a statistical level of 10-4.In black is the corresponding distribution for the genome-wide Bonferroni statistical level of 1.69 10-12. The mode of these distributions were 1.84 and 2.83, respectively. By symmetry on the logarithmic scale, only positive ORs are shown. [file 1471-2350-14-36-S2.docx]

**Additional File2 Density of the minimal interactive Odds Ratio that can be detected with a 80% power in the EOVT study**


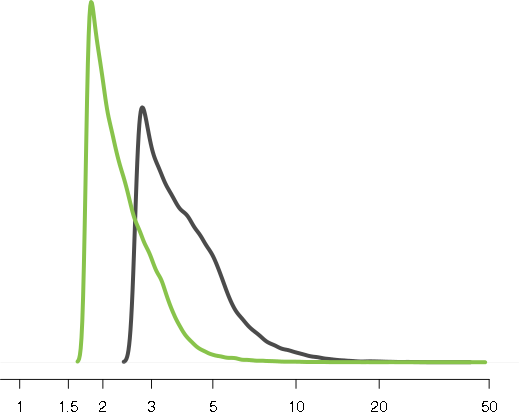


In green is shown the density distribution for a statistical level of 10^-4^.In black is the corresponding distribution for the genome-wide Bonferroni statistical level of 1.69 10^-12^ The mode of these distributions were 1.84 and 2.83, respectively.

By symmetry on the logarithmic scale, only positive ORs are shown.
